# Dietary Exposure to Low Levels of Crude Oil Affects Physiological and Morphological Phenotype in Adults and Their Eggs and Hatchlings of the King Quail (*Coturnix chinensis*)

**DOI:** 10.3389/fphys.2021.661943

**Published:** 2021-04-09

**Authors:** Naim M. Bautista, Lara do Amaral-Silva, Edward Dzialowski, Warren W. Burggren

**Affiliations:** ^1^Zoophysiology, Department of Biology, Aarhus University, Aarhus, Denmark; ^2^Developmental Integrative Biology Research Group, Department of Biological Sciences, University of North Texas, Denton, TX, United States; ^3^Department of Animal Morphology and Physiology, College of Agricultural and Veterinarian Sciences, São Paulo State University, São Paulo, Brazil

**Keywords:** Transgenerational Inheritance, parental effects, crude oil, oxygen consumption, development, epigenetics, physiology, bird

## Abstract

Despite the current knowledge of the devastating effects of external exposure to crude oil on animal mortality, the study of developmental, transgenerational effects of such exposure has received little attention. We used the king quail as an animal model to determine if chronic dietary exposure to crude oil in a parental population would affect morpho-physiological phenotypic variables in their immediate offspring generation. Adult quail were separated into three groups: (1) Control, and two experimental groups dietarily exposed for at least 3 weeks to (2) Low (800 PAH ng/g food), or (3) High (2,400 PAH ng/g food) levels of crude oil. To determine the parental influence on their offspring, we measured metabolic and respiratory physiology in exposed parents and in their non-exposed eggs and hatchlings. Body mass and numerous metabolic (e.g., O_2_ consumption, CO_2_ production) and respiratory (e.g., ventilation frequency and volume) variables did not vary between control and oil exposed parental groups. In contrast, blood PO_2_, PCO_2_, and SO_2_ varied among parental groups. Notably, water loss though the eggshell was increased in eggs from High oil level exposed parents. Respiratory variables of hatchlings did not vary between populations, but hatchlings obtained from High oil-exposed parents exhibited lower capacities to maintain body temperature while exposed to a cooling protocol in comparison to hatchlings from Low- and Control-derived parents. The present study demonstrates that parental exposure to crude oil via diet impacts some aspects of physiological performance of the subsequent first (F*_1_*) generation.

## Introduction

The impacts of crude oil exposure on wild animal populations have been a subject of study since the 1970s when major oil spills first appeared. Oil spills such as The Deepwater Horizon (DWH) extended from the pelagic zone in the ocean to estuaries along the ocean’s coast potentially impacting animal life negatively (Michel et al., 2013; Pasparakis et al., 2019). The weathering process separates crude oil into different phases with different physicochemical characteristics, leading to obvious harmful effects on aquatic species (Dubansky et al., 2013; Tierney et al., 2013; Mager et al., 2014). However, crude oil also contacts shorelines, where sediments remain oil-laden (Pennings et al., 2014; Dubansky et al., 2018). Consequently, oil represents a constant threat for marine and especially marsh species of birds that could be exposed dermally, orally and/or via oil vapor inhalation (Horak et al., 2017; Perez et al., 2017; Harr et al., 2017a, b; Dubansky et al., 2018).

Crude oil exposure in avian species has acute lethal effects on heavily exposed individuals (Perez et al., 2017). Short of proving lethal, however, external (dermal) oil-contamination and respiratory exposures in birds induces increased lung-epithelial CYP1A expression, skin irritation, conjunctivitis and corneal ulcers, and also reduces the feathers’ capacity to repel water, which leads to increased heat loss, and reductions in insulating capacities, buoyancy, and flight performance (Leighton, 1993; Jenssen, 1994; O’Hara and Morandin, 2010; Munilla et al., 2011; Fiorello et al., 2016; Maggini et al., 2017b; Dubansky et al., 2018). Beyond these effects of dermal and respiratory oil exposures, after an oil spill many birds continue being orally exposed for months and even for decades to varying concentrations of petroleum leading also for sublethal effects (Alexander et al., 2017; Esler et al., 2018). Oral exposure to oil occurs via ingestion of contaminated prey, grit and water during foraging, and also occurs during attempts for cleaning (preening) of oil-contaminated feathers (Hartung and Hunt, 1966; Pritsos et al., 2017b). Dietary oil exposure has been reported to induce effects on birds at different levels of biological organization and different developmental stages. For example, loss of body mass has been reported in herring gulls, laughing gull, and kestrels (Pattee and Franson, 1982; Leighton, 1986; Puddephatt, 2014; Horak et al., 2017). Morphological changes and inflammation of the kidney have been reported in mallard ducklings after dietary oil-exposure (Szaro et al., 1980). Hematological responses to dietary oil exposure led to hemolytic anemia, as indicated by increased numbers of Heinz bodies and degenerated erythrocytic organelles in exposed organisms (Pattee and Franson, 1982; Horak et al., 2017; Fallon et al., 2018). Additionally, dietarily oil exposed laughing gulls and cormorants exhibited increased oxidative stress in the liver (Horak et al., 2017; Pritsos et al., 2017a). With respect to physiological effects, potential impairment of blood oxygen carrying capacity was reported in sandpipers, cormorants, homing pigeons, and laughing gulls (Dean et al., 2017; Bursian et al., 2017a). Overall, the results from these studies suggest that dietary/oral exposure to crude oil has significant metabolic implications for bird populations. To our knowledge, this are the first measurements of metabolic rate in oil-exposed birds, which opens a new avenue for exploring the physiology of avian toxicant exposure.

Remarkably, studies aiming to understand the physiological effects of dietary exposure to crude oil in oil-exposed birds – and especially the transgenerational effects on the physiology of their offspring – are scarce. Exposure to crude oil, or to its individual components, induces parent-to-offspring transfer of effects in fish, even when the *first* (*F_1_) generation* itself has not experienced exposure (Corrales et al., 2014; Bautista et al., 2020). Consequently, it is possible that petroleum exposure on bird parental populations could potentially lead to developmental effects in their subsequent generations. Performing this type of transgenerational study on wild birds is rarely practical because of the large amounts of animal rearing space and the time required for birds to develop. Not surprisingly, researchers have turned to the use of animal models or domesticated species (Guerrero-Bosagna et al., 2018; Burggren, 2020).

In the present study, we have used the king quail, Coturnix *chinensis*, as a proxy species to evaluate the effects of dietary exposure to crude oil on the parental P*_0_* generation and subsequent effects on their eggs and hatchlings comprising the F*_1_* generation. First, we determined whether the P*_0_* generation *per se* was affected by assessing multiple physiological and morphological traits. Then, we assessed physiological and morphological traits of F*_1_* eggs and hatchling king quail. We hypothesized that chronic exposure to low levels of crude oil in adult king quail will alter the morphology and physiology of both the P*_0_* adults and their F*_1_* offspring.

## Materials and Methods

### Ethics Statement

All experiments in this study were approved and performed in strict accordance with the Institutional Animal Care and Use Committee (IACUC-Protocol #18022) at the University of North Texas.

### Animal Husbandry

Fifteen pairs of adult king quail *Coturnix chinensis* were obtained from a local supplier (Elm Ridge Enterprises, Temple, United States) and kept at the avian facilities at the University of North Texas. All birds were maintained at ∼24°C and ∼60% humidity in a 14:10 h light:dark cycle, and provided with food and water *ad libitum*. *C. chinensis* exhibit monogamous reproductive behavior. Thus, one male and one female (five pairs per experimental group) were kept together in a ventilated plastic cage that was 60 cm long × 48 cm wide × 35 cm deep. The bottom of the containers were covered with wood shavings to prevent injuries in the feet of the birds and to absorb moisture (MacDonald, 2010; Puddephatt, 2014). Cleaning of the containers was performed on a weekly basis, but all containers were checked, stocked, and cleaned (if necessary) every morning and afternoon.

Eggs collected from the adults’ cages were placed inside a cycle-rotating incubator (GQF, 3258N Digital Command Center, Georgia, United States) and maintained at 37.5°C and 60% RH. King quail eggs take approximately 16 days to hatch (MacDonald, 2010). On day 13 the eggs were removed from the rotation motion of the incubator and placed, until hatch, into individual cells of a custom-built hatcher at 37.5°C and 60% relative humidity. Hatchlings were individually labeled by attaching a piece of tape (<1 cm^2^) to the feathers on the back and transferred into an environmental chamber with the bottom coated with wood flakes to prevent feet injury and absorb moisture. The hatchlings were maintained in similar conditions as in the hatcher under 14:10 h dark/light cycle and provided with water and food *ad libitum*, until seven to 8 days post hatching (dph), at which time respirometry measurements were made (see below). Eggs laid before at least 21 days of parental exposure were not incubated for respirometry nor hematological measurements.

### Diet Preparation

Dietary exposure to crude oil in adult quail was used as the stressor in this study. To prepare oiled diets, solutions of High Energy Water Accommodated Fractions of crude oil (HEWAF) – essentially oil blended into water – were prepared following standard protocols (Mager et al., 2014; Forth et al., 2017; Reddam et al., 2017; Bautista et al., 2019). Source oil “B” (SOB) sampled from the Gulf of Mexico MC252 well on May 22–23, 2010 was used for this experiment. British Petroleum acknowledges the use of a defoamer (Nalco EC9323A), oxygen scavenger (Nalco VX9831), and methanol during the collection of this type of crude oil. Although their presence in our SOB cannot be dismissed, the direct sampling of oil from the riser insertion tube reduces the possibility of incorporation of these compounds into the oil (de Soysa et al., 2012). HEWAF preparation was performed by adding 1,000 mg of crude oil into 1 L of deionized water and blending for 30 s in a commercial blender (Waring TM CB15, Torrington, United States). After blending, the mixture was placed into a separation funnel for 1 h, after which 100 mL of the solution was taken out through a bottom port of the funnel and discarded. 600 mL of the remaining mixture (considered as 100% HEWAF) and two diluted solutions, 1 and 10% HEWAF, in deionized water were used for diet preparation.

Three dietary treatments were used for adult quail (P*_0_*) exposures: (1) Control group, (2) “Low” exposure (1% HEWAF), and (3) “High” exposure (10% HEWAF). To make these dietary treatments, 100 *g* of quail food was prepared from equal parts of 24% protein chick starter (Dumor poultry #5078197, St. Louis, United States), Reptile diet (Mazuri 006267, Gentofte, Denmark), and wild bird mixed seeds (Blain’s Farms and Fleet, Janesville, United States). This mixture was then evenly distributed across the bottom of aluminum trays (100 cm long × 30 cm wide). The food mixture was sprayed with 60 mL of water (Control), or with 60 mL of one of the two HEWAF solution concentrations described above. The treated food was allowed to dry for ∼12 h. The dried food was then collected from the trays and stored at room temperature in glass bottles (Bautista et al., 2019).

Representative samples of each treatment diet were analyzed by ALS Environmental (ALS Environmental, Kelso, United States) to obtain total polycyclic aromatic hydrocarbons (PAH) concentrations (ΣtotPAH). PAHs are petroleum components affecting morphology, cardiac system physiology, flying and swimming performance of vertebrates (Harr et al., 2017b, c; Incardona and Scholz, 2018; Perrichon et al., 2018; King et al., 2020). Thus, determination of [PAH] in dietarily experimental treatments is widely accepted as a valid proxy indication of toxicity level of each treatment in experimental protocols involving animal exposures (Mager et al., 2014; Incardona and Scholz, 2018; Bautista et al., 2020). In addition to dietary samples, eggs obtained from each parental group were also sent for PAH determination analysis to ALS Environmental. Total sum of PAH concentrations in the dietary treatments was 0, 800 and 2,400 ng/(g of food) for the Control, Low and the High exposed groups diets, respectively, (Supplementary Figure 1). These doses are below concentrations used in studies with wild marine species (for a review, see King et al., 2020). In contrast, none of the egg samples from the parental groups exhibited any detectable content of PAHs after analysis (Supplementary Table 1).

### Exposure Protocol

**FIGURE 1:**
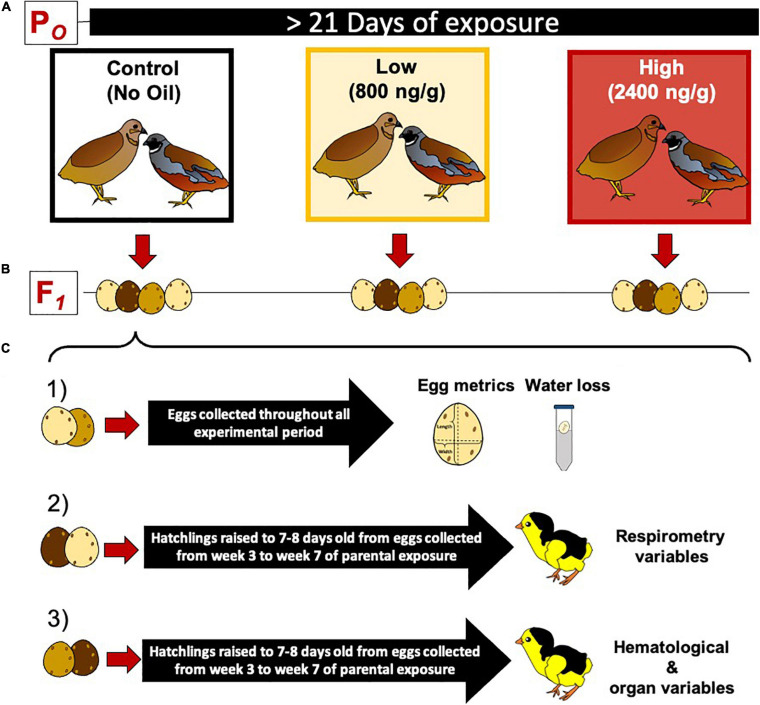
Experimental protocol for dietary crude oil exposure and breeding in the king quail (*Coturnix chinensis*). **(A)** A parental population of adult birds was divided into three groups (5 female and 5 male per group) and exposed to crude oil via diet for at least 21 days. **(B)** Adult quail were maintained as female and male couples (monogamous species) and allowed to breed daily. **(C)** The eggs obtained from each parental group were divided into three subgroups as follows: (1) eggs used to determine egg metrics and water loss, (2) eggs raised to 7–8 day old hatchlings used to measure respirometry variables, and (3) eggs raised to 7–8 days old hatchlings and used to measure blood chemistry, hematology, and organ masses. Eggs used for respirometry and hematological/organ analysis were collected from the third week of exposure toward the end of the experimental time.

Adult quail were allowed to acclimate for 14 days prior to dietary exposure. Before the beginning of exposure, all birds were screened to ensure healthy condition. Dietary exposures began at day 15 of the experiment. Food and water were provided *ad libitum*. Eggs variables were determined throughout all the experimental weeks, however, analysis of respirometry variables in the first generation was performed in hatchlings obtained from eggs collected from adult quail dietarily exposed to crude oil for at least 21 consecutive days (3 weeks; Figure 1). Feeding with control or crude oil-treated food continued until the seventh week of the experiment when the desired number of F*_1_* offspring was obtained. None of the dietary treatments induced any mortality in the experimental P*_0_* groups.

### Assessments of Parental Population (P*_0_*)

Body mass (*g*) was measured by placing each bird on a top pan balance (Mettler Toledo, XA105 DualRange, Columbus, United States). Body mass was recorded at the end of the acclimation period, on weeks 1, 2, 3 5, and 7 and every time that oxygen consumption measurements were made.

#### Respirometry

Measurements of oxygen consumption ($\dot{\text{V}}$O_2_) and carbon dioxide production ($\dot{\text{V}}$CO_2_) of all adult quail were recorded at the end of the acclimation period, before any dietary exposure, and on weeks 1, 2, 3 5, and 7 after the start of the exposure. $\dot{\text{V}}$O_2_ and $\dot{\text{V}}$CO_2_ were measured by flow-through respirometry as previously reported (Amaral-Silva et al., 2020; Sirsat and Dzialowski, 2020). In brief, individual adult quail were placed inside 500 mL opaque respirometers that were maintained in an incubator to control temperature at 24 ± 0.5°C. All individuals were allowed to acclimate to the respirometer for at least 30 min before measurements, during which time a flow of humidified air was maintained through the respirometer. Up to three respirometers were operated simultaneously and individuals from different experimental groups were randomized to minimize effects due to any variation between respirometers. A gas mixture of 20.8–21.2% O_2_, balanced with N_2_ generated with a gas mixer (Model 0154 Brooks Instruments, Hatfield, PA, United States) was delivered as the inflow gas to each respirometer and maintained at a flow rate of ∼370 mL^∗^min^–1^. The flow of the gas mixture was controlled with a flow controller Model 5850E (Brook Instruments Hatfield, United States) and measured with a mass-flow meter FlowBar1 (Sable Systems, Las Vegas, United States). The gas from the outlet of the respirometers was passed through Drierite (Sigma Aldrich, St. Louis, United States) to absorb any humidity before gas analysis and then pulled through the gas analyzers using a flow controller R1 (AEI Technologies, Bastrop, TX, United States). O_2_ and CO_2_ concentrations in outflow respirometer gas were measured using a FC-1B O_2_ and a CA-10 CO_2_ analyzer, respectively, (Sable Systems Las Vegas, United States). Sequential individual sampling of the respirometers was performed for five min using a custom built Arduino based four-channel solenoid multiplexer controlled by LabChart V.7 (ADInstruments, Colorado Springs, United States). Respirometry data were calculated and recorded using LabChart V.7 and Powerlab 16SP (ADInstruments, Dunedin, New Zeland). Two or three measurements per individual quail were averaged to generate $\dot{\text{V}}$O_2_ and $\dot{\text{V}}$CO_2_ values used for analysis.

Cloacal temperature (T*_*b*_*) of each quail was immediately determined after respirometry measurements using a silicon-coated thermocouple inserted approximately 2 cm into the bird’s cloaca. The birds were checked for any signs of injury (not observed in any bird) and returned to their respective housing containers.

$\dot{\text{V}}$O_2_ and $\dot{\text{V}}$CO_2_ (mL^∗^kg^–1*^min^–1^ at standard temperature and pressure in dry conditions, STDP) were calculated with equations Eq. (1) and Eq. (2), respectively, (Lighton, 2018):

$$\dot{V}\,O_{2}={\dot{V}}_{I}*\left(\frac{\left(F_{I}-O_{2}-F_{E}\,O_{2}\right)-F_{E}\,O_{2}\,\left(F_{E}\,C\,O_{2}-F_{I}\,C\,O_{2}\right)}{\left(1-F_{E}\,O_{2}\right)}\right)$$

$$\dot{V}\,C\,O_{2}={\dot{V}}_{I}*\left[\left(\frac{(F_{E}CO_{2}*\left(1-F_{I}O_{2}-F_{I}CO_{2}\right)}{\left(1-F_{E}\,O_{2}-F_{E}\,C\,O\,2\right)}\right)-F_{I}\,C\,O_{2}\right]$$

where *$\dot{\text{V}}$_*I*_* = incurrent flow rate (mL^∗^min^–1^), F*_*I*_*O_2_ = incurrent O_2_ fraction of dry gas, F*_*I*_*CO_2_ = incurrent CO_2_ fraction of dry gas, F*_*E*_*O_2_ = excurrent O_2_ fraction of dry gas, and F*_*E*_*CO_2_ = excurrent CO_2_ fraction of dry gas. After calculating $\dot{\text{V}}$O_2_ and $\dot{\text{V}}$CO_2_, the respiratory exchange ratio (RER) was calculated as $\dot{\text{V}}$CO_2_/$\dot{\text{V}}$O_2_.

Pulmonary ventilation ($\dot{\text{V}}$*_*E*_*) was estimated by plethysmography, using a FD141 pressure transducer-based spirometer (ADInstruments Dunedin, New Zeland) connected to each respirometer. Breathing frequency (*f*, breaths^∗^min^–1^) was determined with the cycle measurements function of LabChart 7 based on the changes in the pressure waves of the recordings. Tidal volume (V*_*T*_*) was calculated as previously reported (Drorbaug and Fenn, 1955; Amaral-Silva et al., 2020; Sirsat and Dzialowski, 2020). Volume calibration of the system was performed by comparison with changes in pressure produced by injection of 250 μl volumes of air into the chambers using a Hamilton glass syringe (Hamilton, Reno, United States; Szdzuy and Mortola, 2007). Relative humidity in the chambers was determined with a humidity sensor (HIH 4021, Honeywell, Minneapolis, United States). Minute ventilation ($\dot{\text{V}}$*_*E*_*) was then calculated from tidal volume and breathing frequency as $\dot{\text{V}}$*_*E*_* = V*_*T*_* ^∗^ *f*. Air convection requirement was calculated as $\dot{\text{V}}$_*E*_/$\dot{\text{V}}$O_2_.

#### Blood and Organ Sampling

At the end of the crude oil exposure period, adult quail were initially anesthetized by placing them into a sealed container with a cotton ball soaked with isoflurane. Anesthetized birds were placed in a dissection tray, and the head of the bird was inserted into a custom -built mask connected to a vaporizer to maintain constant flow of isoflurane (1.5% in air) vapors to maintain light anesthesia. An incision was made at the top of the breast area to obtain an unobstructed view of the heart under the sternum. Blood sampling and analysis was performed as described elsewhere (Tazawa et al., 2002). Briefly, blood samples of 400 ± 50 μl were obtained directly from the right brachiocephalic artery using a one ml syringe previously heparinized. Sampled blood was placed in sterile 1.5 mL centrifuge tube. Blood collection was performed within a 1-min sampling period. Immediately after collection, blood samples were processed and analyzed, in the following sequence: (a) 120 μl of blood were analyzed for pH, partial pressure of oxygen (PO*_2_*, mmHg), partial pressure of carbon dioxide (PCO*_2_*, mmHg), blood oxygen saturation (SO_2_, %), and bicarbonate concentration ([HCO^–^_3_], mmol/L) using a Radiometer blood gas analyzer ABL5 (Diamond Diagnosis, Holliston, United States); (b) 10 μl were used to estimate mean corpuscular hemoglobin concentration ([MCHb], g%) using an hematology analyzer (Beckman Coulter *diff*, Indianapolis, United States); (c) 10 μl were used to estimate osmolality with a vapor pressure osmometer (Vapro 5520, EliTech, Puteaux, France); (d) 10 μl were used to estimate lactate concentration (mmol/l) with a lactate meter (Nova biomedical – 39654, Waltham, United States); (e) and 140 μl of blood were used to measure hematocrit (Hct, %) in duplicate using 70 μl capillary tubes (Thermo Fisher Scientific, 22-362-566 Waltham, United States) and spun for 5 min in a micro hematocrit centrifuge (LW Scientific M24, Lawrenceville, United States). With the exception of hematocrit, all measurements were obtained within 2 min after blood was sampled.

Immediately after blood extraction, the deeply anesthetized birds were euthanized by decapitation. The major organs (brain, eyes, lungs, heart, liver, spleen, kidneys, stomach and intestines, and gonads) were excised and weighed to the closest mg using a micro balance (XA105 DualRange, Mettler Toledo, Columbus, United States).

### Assessments of Offspring Population (F*_1_*)

#### Egg Variables

All adult housing containers were daily checked for newly laid eggs 2 h after the beginning of the light cycle during the morning and 2 h prior to the dark cycle during the evening. Collected eggs were labeled and immediately weighed on a digital top-loading balance (Symmetry EC-series, Cole Parmer, Vernon Hills, IL, United States) to the closest mg. Egg length and width were obtained to the nearest mm using a digital caliper. Egg mass, length, and width were determined throughout the experiments. Offspring eggs obtained from each parental group (control, Low HEWAF and High HEWAF diets) were divided into one of three different experimental protocols (Figures 1B,C).

#### Egg Water Loss

The first egg group (Figure 1C.1) was used to measure water loss (W_*loss*_, mL/day) through the eggshell, determined by measuring the egg mass changes, representing water loss, over time. Mass of the intact egg was determined immediately after collection, and then the egg was immediately placed into a sealed 50 mL Falcon tube filled with the desiccant Drierite (Sigma Aldrich St. Louis, United States) up to the 25 mL mark and the egg was left undisturbed for 24 h. After this period, water loss throughout the eggshell was calculated as the difference in egg mass before and after the 24 h desiccation period (Paganelli, 1980; Ackerman and Rahn, 1981; Spotila et al., 1981).

#### F1 Hatchling Respirometry

The second group of eggs was used in metabolic measurements. $\dot{\text{V}}$O2 and $\dot{\text{V}}$CO2 were measured in 7–8 dph old F1 hatchlings to determine if parental dietary crude oil exposure had an effect on offspring metabolic traits. $\dot{\text{V}}$O_2_ and $\dot{\text{V}}$CO_2_ were determined in the F*_1_* under a cooling protocol to assess if physiological variables associated with thermogenic capacities were affected by the parental exposure. F*_1_* quail offspring from the three parental populations were deprived from food 1 h prior to start of the $\dot{\text{V}}$O_2_ measurement. Each hatchling was individually placed into a ∼250 ml metal respirometer with an inlet and outlet port affixed in the lid of the chamber. Respirometer temperature was controlled by placing the respirometer into a 37°C incubator and the birds were allowed to acclimate within the respirometer for a 45 min period. Body temperature (T*_*b*_*) was recorded during measurements using a silicon coated thermocouple adapted with a plastic base that facilitated attachment to the feathers, passing through the lid of the chamber, inserted into the hatchling’s cloaca (∼1 cm), and glued to the surrounding feathers. $\dot{\text{V}}$O_2_ and $\dot{\text{V}}$CO_2_ were estimated using the same equipment arrangement as for the parental population described above. After the acclimation period, chamber temperature was gradually decreased at a rate of 3°C every 30 min (Swanson and Liknes, 2006), until reaching 19°C. Two – three measurements per individual were obtained at each temperature step and averaged for analysis. Additionally, pulmonary ventilation ($\dot{\text{V}}$*_*E*_*), *f*, V*_*T*_* and air convection requirement for the F_1_ hatchling groups were calculated as described above for the parental populations. After $\dot{\text{V}}$O_2_ and $\dot{\text{V}}$CO_2_ measurements were finished, the hatchlings were deeply anesthetized with isoflurane by placing them into a sealed container, and then decapitated.

#### F_1_ Hatchling Blood and Organ Sampling

The third group of eggs was incubated and maintained until 7–8 dph as described above (Figure 1C). At this stage, the hatchlings were anesthetized by placing them into a sealed container with a cotton ball soaked with isoflurane. Anesthetized hatchlings were placed in a dissection tray, and the head of the bird was inserted into a custom -built mask connected to a vaporizer to maintain constant flow of isoflurane (1.5% in air) vapors to maintain anesthesia. Similar to the parental population, an incision was made at the top of the breast area to obtain an unobstructed view of the heart under the sternum and blood was withdrawn from the brachiocephalic artery using a previously heparinized syringe. Hematological variables were measured as described above for the parental population. Because of the small size of the animals (∼8.1 *g*) the maximum blood sample size was 300 μl. After blood sampling, the offspring quail were immediately decapitated while still under anesthesia and all major organs were excised, weighed and fixed. An attempt was made to determine the sex of each hatchling during dissection of the organs, however, the stage gonadal development in 7–8 dph king quails do not allowed for determination of the sex with 100% certainty, therefore, hatchlings sex was not considered in statistical analysis.

### Statistical Analysis

#### Parental Population

Statistical analysis and graphing were performed on R software version (4.0.3) and Rstudio (1.4.1106), and Prism V.8 (GraphPad), respectively. Normality of the residuals was interpreted from graphical analysis and from statistical output of Shapiro–Wilk test. Homogeneity of variance in residuals was analyzed with Levene’s homogeneity test. Adult body mass and T*_*b*_* were compared among groups using a general linear model (GLM) from the lme4 R package, in which experimental group (Control, Low, or High exposure level), sex, and experimental period (time in weeks) were used as fixed effects. Similarly, $\dot{\text{V}}$O_2_, $\dot{\text{V}}$CO_2_, RER, $\dot{\text{V}}$*_*E*_*, V*_*T*_*, *f*, and $\dot{\text{V}}$*_*E*_*/$\dot{\text{V}}$O_2_ were compared among groups using a similar GLM model, however, body mass and T*_*b*_* of the quail were considered as covariates.

Hematological variables of the parental population were compared between groups with GLM model using experimental group and the sex as fixed effects, and considering body mass and body temperature as covariates. Similarly, organ masses were analyzed with a similar mode as for blood variables, but T*_*b*_* was not considered as covariate. Organ mass data were expressed as percentage of body mass.

#### Offspring Population (F_1_)

Egg mass, egg length, and egg width, were compared among F*_1_* offspring groups with GLM model considering experimental group and experimental time as fixed effects. Egg W_*loss*_ was compared between groups considering experimental group and experimental time as fixed effects and initial egg mass as covariate.

Body mass of hatchling quails was compared between experimental groups considering week of collection as random effect. T*_*b*_*, $\dot{\text{V}}$O_2_, $\dot{\text{V}}$CO_2_, RER, $\dot{\text{V}}$*_*E*_*, V*_*T*_*, *f*, and $\dot{\text{V}}$*_*E*_*/$\dot{\text{V}}$O_2_ were analyzed using repeated measurements mixed effects model considering experimental group and cooling temperature as fixed effects, body mass as covariate, and week of collection as random effect.

Hematological variables in offspring population were compared between groups considering body mass and body temperature as covariates and week of collection as random effect. Finally, organ masses in the offspring generation were compared among groups considering body mass as covariate and week of collection as random effect.

The *a posteriori* Tukey’s honest significant difference (HSD) test was used to determine mean differences. In addition, when one specific factor induced significant effects on the analyzed variable, all pairwise multiple comparisons were performed with Holm-Sidak procedure to explore differences between the different levels within the factor. Statistical significance was considered with a *p* value < 0.05 and α value of 0.05. Data are expressed as mean ± s.e.m, unless otherwise stated.

## Results

### Parental Population

None of the dietary treatments induced any mortality in the experimental groups. Moreover, we did not observe changes in appetite or notice any difference in the behavior of the birds. However, the experimental group fed with the High dietary treatment diet exhibited moderate diarrhea.

#### Adult Body Mass

Crude oil exposure in either amount had no significant effect (*P* > 0.05) on adult body mass for either males (range 44.5 to 45.8 *g*) or females (range 58.7 to 63.8 *g*) across all experimental weeks. However, body mass was significantly lower in males compared to females at all measurement points (*P* < 0.001).

#### Adult Body Temperature (T_*b*_)

Similarly to body mass, T_*b*_ of the adult populations, which averaged 40.7 ± 0.18°C, was also not significantly different between sexes in any groups (*P* = 0.193).

#### $\dot{\text{V}}$O_2_ and $\dot{\text{V}}$CO_2_ in the P_*O*_ Adults

**FIGURE 2:**
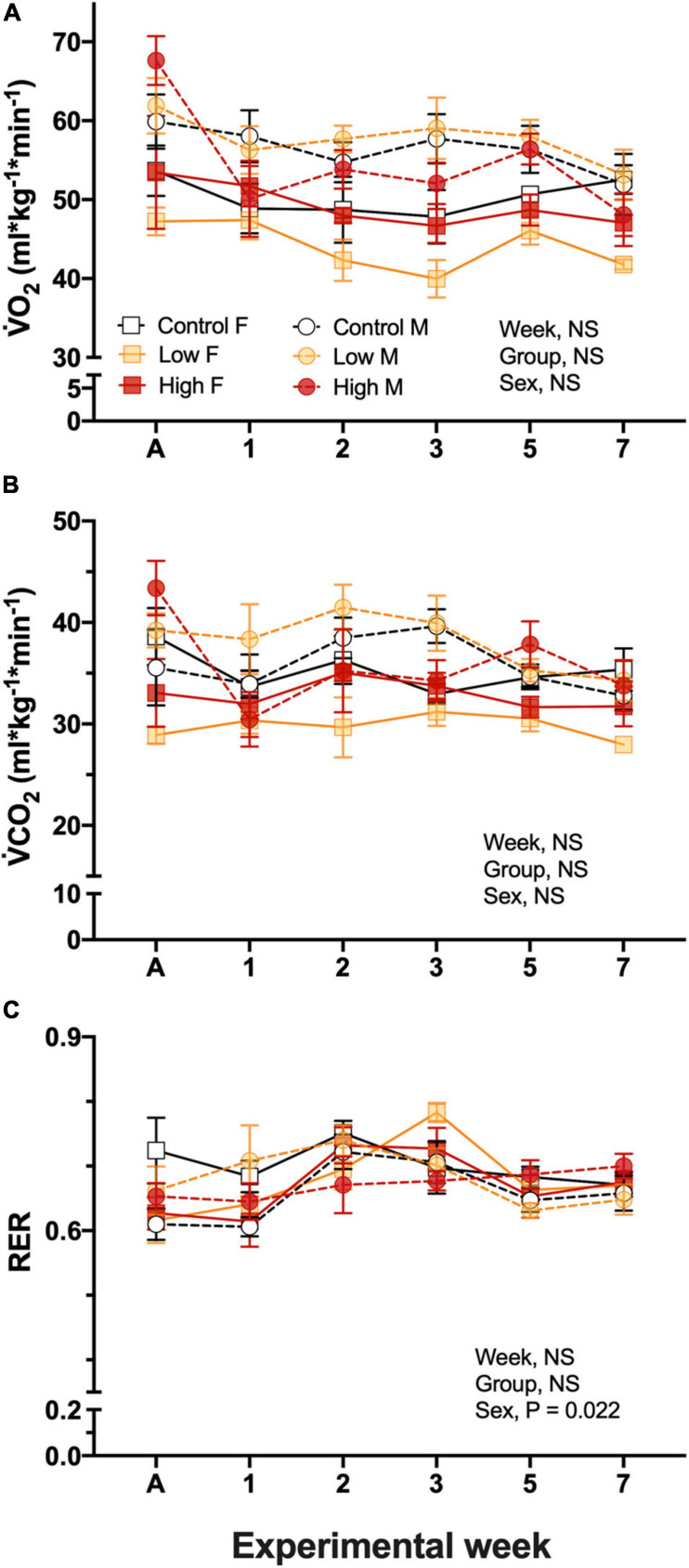
Metabolic variables during experimentation in female (squares), and male (circles) groups of parental populations of king quail dietarily exposed to Low (1%HEWAF, light yellow symbols), High (10% HEWAF, red symbols), and Control (white symbols). **(A)** Oxygen consumption ($\dot{\text{V}}$O_2_), **(B)** Carbon dioxide production ($\dot{\text{V}}$CO_2_), and **(C)** Respiratory exchange ratio (RER). Data are expressed as mean ± 1 standard error of the mean. Statistical significance was considered to be α = 0.05. *n* = 3–5 per data point.

Resting $\dot{\text{V}}$O_2_ in the parental populations was ∼50–60 mL O_2_.kg.min^–1^, and was not significantly affected by experimental dietary treatment, quail sex, nor experimental week, and there was not significant interactions among the factors (Figure 2A). $\dot{\text{V}}$O_2_ was not related to T*_*b*_*, however, it was significantly related to body mass of the quail (*P* = 0.0005). Similar to $\dot{\text{V}}$O_2_, none of the considered factors affected $\dot{\text{V}}$CO_2_ values and were maintained in a range between 28.9 ± 0.9 and 41.5 ± 2.2 mL/kg/min, similarly to $\dot{\text{V}}$O_2_ this variable was also significantly related to body mass (*P* = 0.0015, Figure 2B).

Respiratory exchange ratio, RER calculated by the ratio of $\dot{\text{V}}$CO_2_/$\dot{\text{V}}$O_2,_ ranged between 0.61 ± 0.04 and 0.75 ± 0.02 and no interactions were found among the factors. Additionally, neither exposure treatment nor experimental period had an effect on this variable, however, the sex of the quail had an individual effect (*P* = 0.022). RER varied with experimental week, but no clear pattern of change emerged (Figure 2C).

**FIGURE 3:**
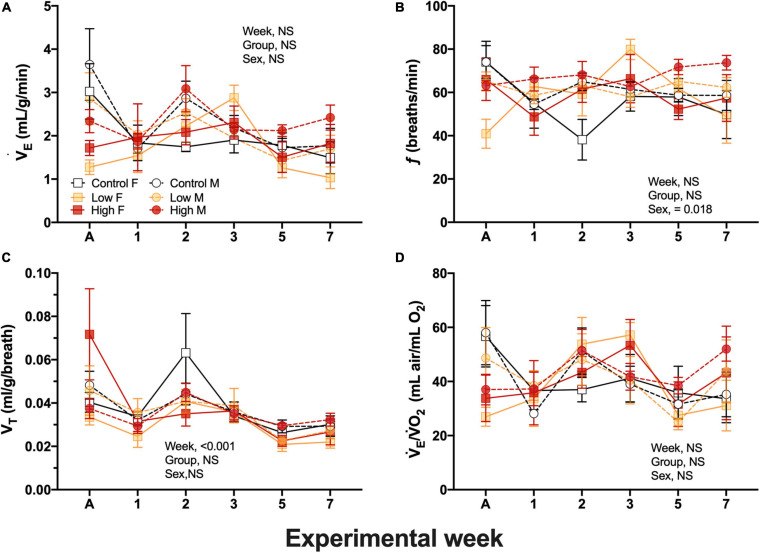
Ventilation variables during experimentation in adult female (squares), and adult male (circles) groups of parental populations of king quail dietarily exposed to Low (1%HEWAF, yellow symbols), High (10% HEWAF, red symbols), and Control (white symbols). **(A)** Minute ventilation ($\dot{\text{V}}$*_*E*_*). **(B)** Breathing frequency (*f*), **(C)** Tidal volume (V*_*T*_*). **(D)** Air convection requirement ($\dot{\text{V}}$*_*E*_*/$\dot{\text{V}}$O_2_). Data are expressed as mean ± 1 standard error of the mean. Statistical significance was considered to be α = 0.05. *n* = 3–5 per data point.

$\dot{\text{V}}$_*E*_ and the air convection requirement did not differ among experimental groups, the sex of the quail, nor experimental time and ranged between 2.01 ± 0.52 and 1.45 ± 0.23 mL/g/min in female, and between 2.94 ± 0.37 and 1.96 ± 0.22 mL/g/min in males with no clear trend in the results (Figure 3A). Breathing frequency, f, did not differed between experimental groups and ranged from 63.4 ± 2.2 to 73.7 ± 3.4 breaths/min in males and 40.9 ± 6.7 to 79.8 ± 4.7 breaths/min in females during experimentation (Figure 3B). f did not significantly differ between sex (*P* > 0.05). Similarly, tidal volume did not significantly vary between the sex of the quail (*P* > 0.05) and dietary treatment (*P* > 0.05), but V_*T*_ varied significantly with experimental week (*P* < 0.018) without clear trend (Figure 3C). V_*T*_ values ranged from 0.048 ± 0.011 to 0.026 ± 0.002 mL/g/breath in female and in 0.043 ± 0.003 to 0.029 ± 0.001 mL/g/breath in male quail (Figure 3C). Similarly to V_*T*_, the air convection requirement did not varied based on dietary treatment, sex, week, or neither of the covariates. $\dot{\text{V}}$*_*E*_*/$\dot{\text{V}}$O_2_ averaged 40.4 ± 1.7 mL air/g/mL O_2_ across experimental time (Figure 3D).

#### Adult Blood Chemistry and Hematology

Results of the different blood variables measured are summarized in Table 1. Dietary exposure to crude oil induced significant changes in three of the measured blood parameters. The Control group, both female and male birds, exhibited the lowest PCO_2_ mmHg values in their blood (24.5 ± 0.3 and 27 ± 1.4 for male and female, respectively) when compared to the Low (27.4 ± 2.3, 27.6 ± 1.6) and High oil (27.4 ± 2.3, 31.2 ± 1.5) exposed groups (*P* < 0.008). Arterial blood PO_2_ was also affected by the dietary treatment, with the Low and High oil-exposed groups exhibiting the higher and lower values, respectively, (88.4 ± 1 and 85.8 ± 1, *P* < 0.02). Oxygen saturation values from oil-exposed groups were significantly lower (∼96%, *P* = 0.0003) in comparison to the Control group (∼97.5%). With exception of Hb (10.7 ± 0.2 g%) and Hct (31.6%) values, where female quail from the High oil-exposed group exhibited significantly lower values in comparison to males, the rest of the measured blood chemistry variables ([HCO_3_^–^], OSM, Lactate, and pH) were not significantly different (Table 1).

**TABLE 1:** Blood chemistry and hematology in the parental adult populations (P*_*O*_*), and in the first generation offspring populations (F*_1_*) of king quail.

		Parental adult population (P*_*O*_*)						Offspring population (F*_1_*)		
	Group	Control		Low oil diet		High oil diet		Control	Low oil diet	High oil diet
	
	Sex	Female	Male	Female	Male	Female	Male			
	*n*	4	4	3–5	5	5	4–5	16–17	20–22	20–21
Arterial blood chemistry	pH	7.42 ± 0.02	7.46 ± 0.04	7.45 ± 0.02	7.43 ± 0.01	7.41 ± 0.02	7.45 ± 0.01	7.43 ± 0.04	7.43 ± 0.02	7.48 ± 0.02
	PCO_2_ (mmHg)	**24.5 ± 0.3^*a*^**	**27 ± 1.4^*a*^**	**27.4 ± 2.3^*b*^**	**27.6 ± 1.6^*b*^**	**31.2 ± 1.5^*c*^**	**32.4 ± 0.9^*c*^**	39.5 ± 3.7	36.2 ± 3.3	31.5 ± 0.8
	PO_2_ (mmHg)	**88.3 ± 0.5^*ab*^**	**87 ± 1.1^*ab*^**	**88.4 ± 1.0^*a*^**	**88.8 ± 0.7^*a*^**	**85.8 ± 1.0^*b*^**	**85.7 ± 0.9^*b*^**	99.5 ± 4.8	99.3 ± 5.1	98.9 ± 3.2
	SO_2_ (%)	**98 ± 0.4^*a*^**	**97 ± 0.4^*a*^**	**96.6 ± 0.7^*b*^**	**95.8 ± 0.9^*b*^**	**94.6 ± 0.2^*b*^**	**95.2 ± 0.5^*b*^**	95.5 ± 1.8	94.0 ± 2.6	97.2 ± 0.5
	HCO^–^_3_ (mmol/L)	23.2 ± 0.5	22.2 ± 1.8	23.8 ± 0.6	22.4 ± 1.0	21.6 ± 0.7	22.8 ± 1.0	22.4 ± 1.1	21.7 ± 0.5	24.5 ± 0.7
	OSM (mmol/Kg^–1^)	305.5 ± 1.4	306 ± 1.4	307 ± 3.2	303.8 ± 0.9	305 ± 1.5	302.4 ± 2.9	297 ± 2.8	298.8 ± 3.2	291.3 ± 2.1
	Lac (mmol/L)	3.3 ± 0.6	3.3 ± 0.6	3.7 ± 1.3	3.94 ± 0.6	4.5 ± 0.6	3.3 ± 0.6	4.1 ± 0.6	4.7 ± 0.5	5.5 ± 0.9
Hematology	Hct (%)	31.8 ± 3.1	33.9 ± 2.9	36.7 ± 1.9	37.7 ± 1.7	*31.6 ± 1.0	40.9 ± 2.4	29.2 ± 2	32.9 ± 0.7	32.3 ± 0.8
	Hb (g%)	11.8 ± 0.6	13.2 ± 0.6	12.2 ± 0.4	11.6 ± 0.5^*a*^	*10.7 ± 0.2	13.5 ± 0.6	10.3 ± 0.3	10.5 ± 0.2	10.4 ± 0.3

Different superscript letters indicate significant differences among experimental groups. “*” in Hct and Hb in the High oil diet group of the parental population indicates significant differences between sex. Significantly different values are also indicated in bold.

#### Organ Mass in the P_0_

Organ masses, expressed as percentage of body mass, are presented in Table 2. Heart mass was smaller in female and male quail in both oil-exposed groups compared to the Control group (Table 2). Kidney mass was higher in the Low-oil exposed group in comparison to the Control and the High-oil exposed group and differed between sex in all experimental groups (Table 2). Liver, digestive tract and ceca masses were not affected by oil exposure, but differences between sexes were found within all experimental groups in which female quail exhibited higher masses of these organs in comparison to males (Table 2). These three variables were related with body mass. Brain mass as a percentage of body mass was also not affected by exposure to crude oil, but in female quail the values were smaller in comparison to the male groups in all three experimental groups. Lungs and eye mass did not differ neither between groups nor on the basis of sex within treatments (Table 2).

**TABLE 2:** Organ masses (% of body mass) in the parental adult population (P*_*O*_*), and in the offspring population (F*_1_*).

	Adult parental generation (P*_*O*_*)						Offspring population (F*_1_*)		
	Control		Low oil diet		High oil diet		Control	Low oil diet	High oil diet
	Female	Male	Female	Male	Female	Male			
Organ/n	4	4	5	5	5	5	8	11	10
Heart	**0.74 ± 0.05^*a*^**	**0.9 ± 0.05^*a*^**	**0.65 ± 0.06^*b*^**	**0.7 ± 0.04^*b*^**	**0.68 ± 0.04^*b*^**	**0.7 ± 0.02^*b*^**	1.2 ± 0.04	1.2 ± 0.04	1.1 ± 0.03
Liver	*3.05 ± 0.42	1.84 ± 0.1	*2.9 ± 0.18	1.92 ± 0.07	*3.02 ± 0.16	1.86 ± 0.11	4.3 ± 0.4	4.4 ± 0.2	4.7 ± 0.3
Lungs	0.87 ± 0.11	0.8 ± 0.07	0.74 ± 0.06	0.88 ± 0.06	0.68 ± 0.03	0.74 ± 0.07	0.7 ± 0.04	0.8 ± 0.04	0.8 ± 0.02
Gut	*2.38 ± 0.16	1.77 ± 0.09	*2.48 ± 0.28	2.18 ± 0.35	*2.7 ± 0.15	1.65 ± 0.18	5.7 ± 0.9	6.3 ± 0.4	6.3 ± 0.5
Eyes	0.53 ± 0.05	0.97 ± 0.12	0.65 ± 0.05	0.8 ± 0.02	0.57 ± 0.02	0.81 ± 0.04	2.5 ± 0.2	2.3 ± 0.12	2.2 ± 0.1
Brain	*0.84 ± 0.11	1.11 ± 0.04	*0.77 ± 0.03	1.03 ± 0.03	*0.73 ± 0.02	1.01 ± 0.03	3.3 ± 0.2	3.2 ± 0.2	2.9 ± 0.1
Ceca	*0.43 ± 0.02	0.4 ± 0.03	*0.82 ± 0.1	0.64 ± 0.15	*0.48 ± 0.06	0.37 ± 0.03	1.0 ± 0.04	1.0 ± 0.08	1.1 ± 0.03
Kidneys	*0.9 ± 0.08^*a*^	**0.67 ± 0.04^*a*^**	*1.03 ± 0.08^*b*^	**0.71 ± 0.03^*b*^**	*0.99 ± 0.03^*a*^	**0.62 ± 0.02^*a*^**	1.3 ± 0.2	1.1 ± 0.08	1.3 ± 0.2

“*” indicates differences between sex within the adult experimental groups. Significantly different values are indicated in bold font and with different superscript letters.

### Offspring Population (F*_1_*)

#### Egg Variables

**FIGURE 4:**
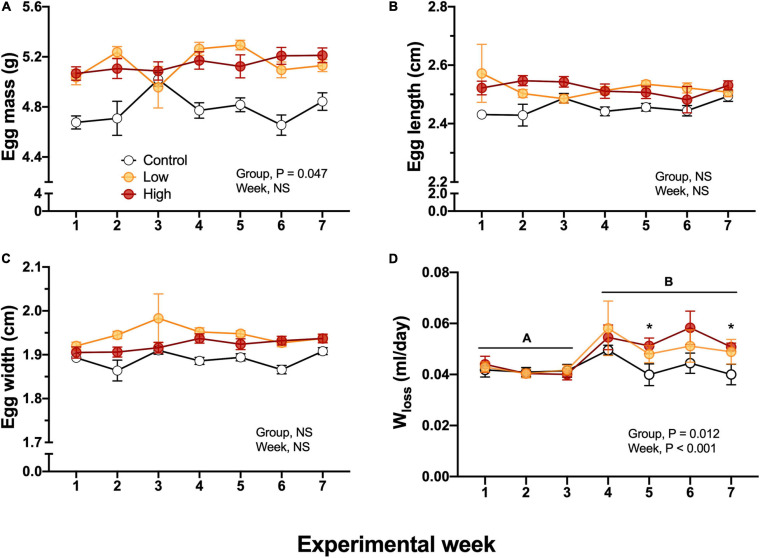
Metrics and water loss in F_1_ eggs obtained from of king quail. **(A)** Egg mass (g), **(B)** Egg length (cm), **(C)** Egg width (cm), and **(D)** Water loss. Data is expressed as mean ± 1 standard error of the mean. Statistical significance was considered with α = 0.05. Different letters indicate statistical differences between weeks. “*” Indicate statistically significant differences between groups at the specific experimental weeks. *n* = 6–29 per data point.

Egg mass obtained from the Low and High concentration groups exhibited higher values (5.2 ± 0.4, 5.1 ± 0.8 *g*, respectively) in comparison with the eggs from the Control parental group (4.8 ± 0.7*g*, *P* = 0.047). These egg mass differences were present from the beginning of the experimental week and were constant throughout all the experiments. Thus, they cannot be attributable to the dietary parental oil exposure (Figure 4A). Egg length (2.5 ± 0.012 cm) and egg width (1.9 ± 0.011 cm) were not significantly different among experimental groups (Figures 4B,C). Egg water loss did not differ between groups during the first 4 weeks of exposure (Figure 4D). However, at week five the eggs from the Control parental group exhibited lower water loss volumes (0.04 ± 0.004 mL/day) in comparison with eggs obtained from Low and High-exposed groups (0.05 ± 0.003, and 0.051 ± 0.003 mL/day). This difference was also observed at the last week of experimental time (Figure 4D).

#### Body Mass

Body mass of 7–8 days old hatchlings did not differ among experimental groups (*P* = 0.287), including when week of collection was used as random effect (*P* = 1.0). Body mass averaged 7.9 ± 0.27, 8.2 ± 0.24, and 8.6 ± 0.25 *g* for Control, Low and High groups.

#### Body Temperature and Metabolism

**FIGURE 5:**
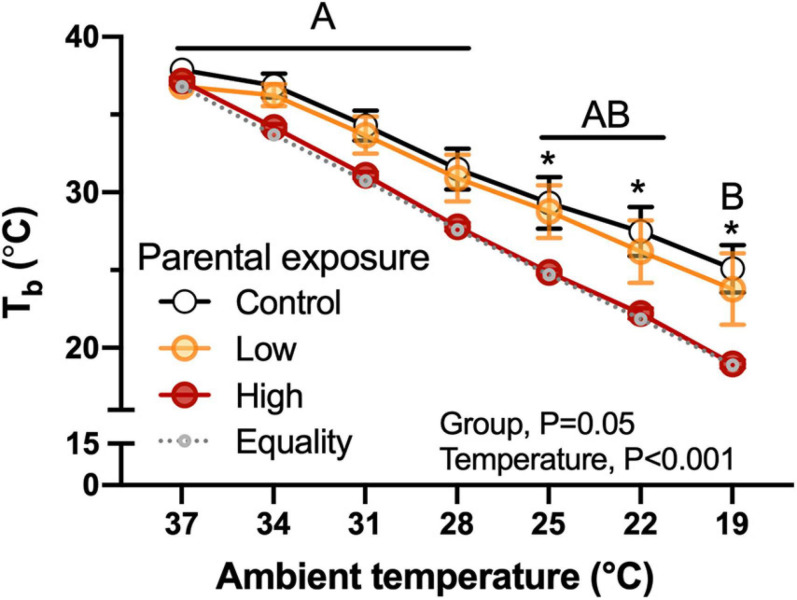
Body temperature (T*_*b*_*) as a function of declining ambient temperature in 7–8 day old king quail hatchlings (F*_1_*) derived from parents (P*_0_*) exposed to Control conditions or Low or High oil exposure through diet. Line of equality representing the decrease in temperature by the cooling protocol is shown as a gray dotted line. Data are expressed as mean ± 1 standard error of the mean. Different letters indicate statistical differences between temperatures. “*” indicate statistically significant differences between groups at the specific temperature. *n* = 4–8 per data point.

Body temperature of hatchlings was not related with body mass and the High-oil-exposed P_0_ exhibited lower values in comparison with the Low or the Control groups (Figure 5). This difference became evident when ambient temperature was reduced to 25°C and was maintained until the end of the experimental test. Similarly to body mass, there were no significant differences (*P* = 1.0) when week of collection was used as a random effect.

**FIGURE 6:**
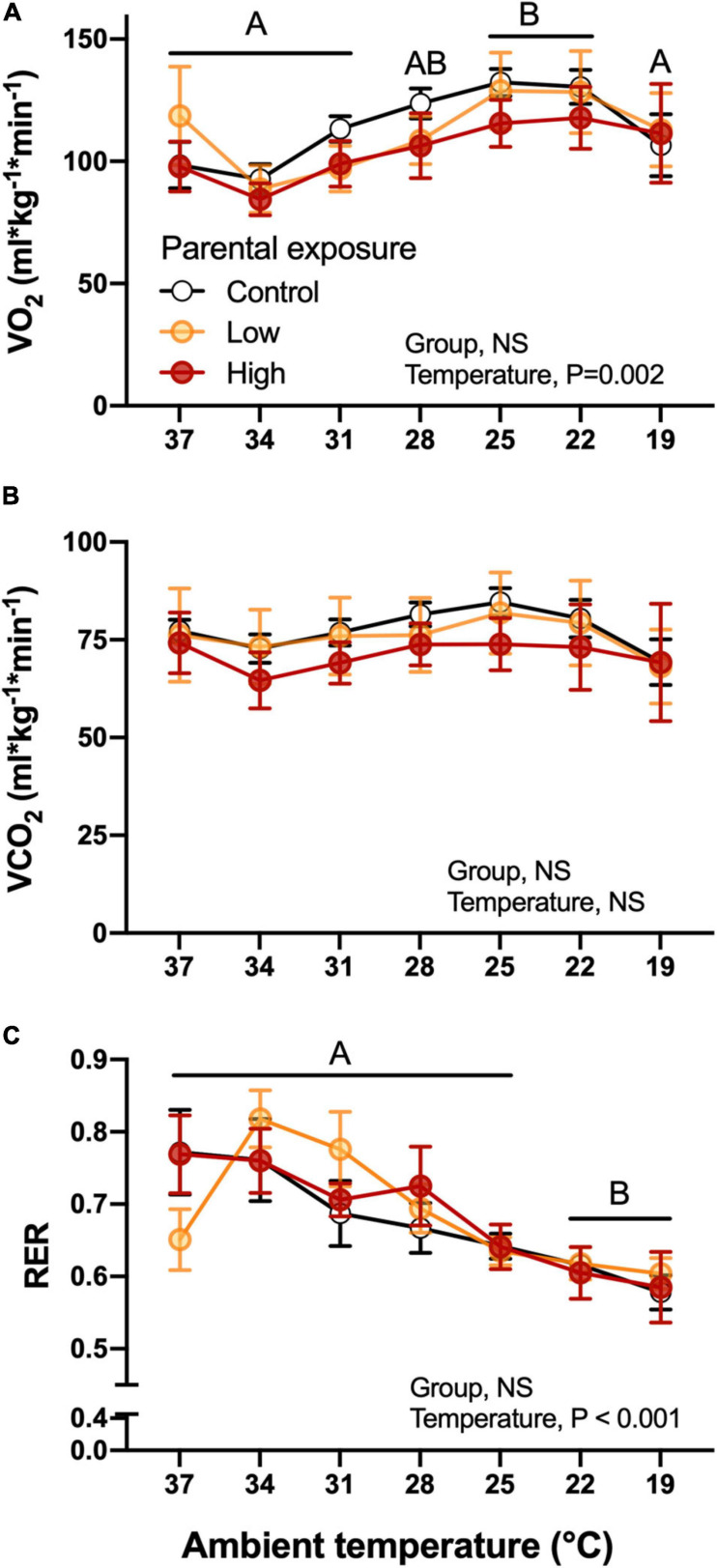
Metabolic variables as a function of declining ambient temperature in 7–8 day old king quail hatchlings (F*_1_*) derived from parents (P*_0_*) exposed to control conditions or Low or High oil exposure through diet. **(A)** Oxygen consumption $\dot{\text{V}}$CO_2_, **(B)** carbon dioxide production ($\dot{\text{V}}$CO_2_), and **(C)** respiratory exchange ratio (RER). Data are expressed as mean ± 1 standard error of the mean. Different letters indicate statistical differences of mean temperature values at specified ambient temperatures in comparison to the 37 temperature values. *n* = 4–8 per data point.

Parental experimental dietary exposure to Low or High levels of oil did not affect $\dot{\text{V}}$O_2_ of the F*_1_* offspring during the cooling challenge (Figure 6A). However, the decrease in temperature induced significant increases in oxygen consumption at 25 and 22°C in the F*_1_* quail in general (Figure 6A). Although $\dot{\text{V}}$CO_2_ followed a similar trend as $\dot{\text{V}}$O_2_ (Figure 6B), the decrease in temperature did not significantly affect this variable (*P* > 0.05). Both of these variables were significantly associated with body mass of the hatchlings. Additionally, RER was not affected by parental exposure, but did change overall as a function of temperature, generally decreasing as the temperature of the challenge was decreased (Figure 6C). There were also no significant differences (*P* > 0.05) when week of collection was used as a random effect.

#### Ventilatory Variables and Body Temperature

**FIGURE 7:**
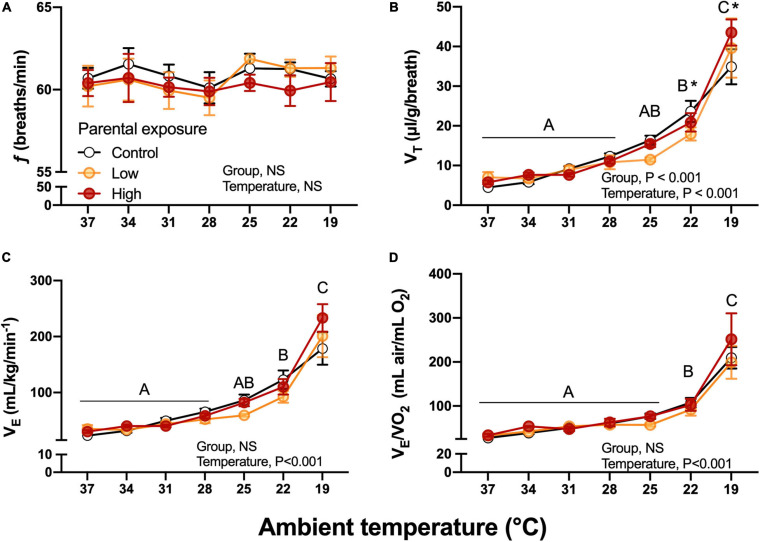
Ventilatory variables as a function of declining ambient temperature in 7–8 day old king quail hatchlings (F*_1_*) derived from parents (P*_0_*) exposed to control conditions or low or medium oil exposure through diet. **(A)** Breathing frequency (*f*). **(B)** Tidal volume (V*_*T*_*). **(C)** Minute ventilation ($\dot{\text{V}}$*_*E*_*). **(D)** Air convection requirement ($\dot{\text{V}}$*_*E*_*/$\dot{\text{V}}$O_2_). Data are expressed as mean ± 1 standard error of the mean. Different letters indicate statistically significant differences between temperatures. “*” indicate statistical differences between the groups at the specified temperature. *n* = 4–8 per data point.

Breathing frequency, *f*, was unaffected by diet or by ambient temperature (Figure 7A). While not affected by parental diet, V*_*T*_* increased during the cooling challenge, reaching a value more than 600% higher than at 37°C (Figure 7B). Unsurprisingly, then $\dot{\text{V}}$_*E*_ also increased from 3.48 ± 0.43 to 23.92 ± 1.49 mL/g/min, a nearly 700% increase. This sharp rise in $\dot{\text{V}}$_*E*_ occurred equally in all groups until 22°C, but at 19°C $\dot{\text{V}}$_*E*_ was lowest in the Control group and highest in the High exposure group (Figure 7C). Finally, air convection requirement followed a similar trend to $\dot{\text{V}}$*_*E*_*, significantly and sharply increasing at 19°C (Figure 7D). V*_*T*_*, $\dot{\text{V}}$_*E*_ and $\dot{\text{V}}$_*E*_/$\dot{\text{V}}$O_2_ were significantly associated with body mass (*P* < 0.05). No significant differences (*P* > 0.05) emerged when the week of collection used as random effect.

#### Blood Chemistry and Hematological Variables and Organ Masses

None of the blood chemistry or hematological variables measured in the offspring population varied significantly between experimental groups (Table 1). With exception of PO_2_ and PCO_2_ values that were associated with Tb of the hatchling, none of the variables were associated with the covariates for any of the hematological variables (the random effect of week of collection was not statistically significant, *P* > 0.05), with the exception of Osm (*P* < 0.002), Noteworthy is the fact that the values obtained in this study for the king quail fall within the ranges previously reported for hatchlings of the bobwhite quail (*Colinus virginianus*), the common quail (*Coturnix coturnix*), and the chicken (*Gallus gallus*; Burggren and Elmonoufy, 2017; Flores-Santin et al., 2018; Amaral-Silva et al., 2020). Similarly, none of the organ masses significantly differed between hatchlings from the Control, Low or High oil-exposed parental groups (Table 2) and, for none of the organ masses when the random effect of week of collection was included, with the exception of gut mass (*P* < 0.002).

## Discussion

Dietary oil exposure induces severe effects on directly exposed adult bird populations. Compounding the problem, oil spills may also coincide with the breeding season of numerous bird species, as was the case for the DWH oil spill (Bernardo, 1996; Vidal et al., 2011; Horak et al., 2017; Perez et al., 2017; Harr et al., 2017a, b). Exposed adult populations have exhibited reductions of clutch size and egg abandonment (Azkona et al., 2006). Thinning of the eggshell was also reported in mallard ducks (Stubblefield et al., 1995). Direct oil exposure on bird eggs has been reported to lead to increased mortality in chickens and gulls (Leighton, 1993). Reduced hatching success has also been reported in mallard ducks (Albers, 1977; Szaro et al., 1980). Along with these effects, crude oil exposure leads to heart, liver, and spleen enlargement in bird embryos (Couillard and Leighton, 1989, 1991). However, beyond the effects that direct exposure to crude oil induce in adult and young bird populations, oil exposure in adults during breeding season could potentially lead to transgenerational effects in their offspring generations. In the present study, we explored whether P*_*O*_* exposure led to phenotype change in their immediate F*_1_* generation that was itself was not exposed to crude oil, by measuring morphological and respiratory-related variables in eggs and hatchlings obtained from the oil-exposed parental populations. To the authors’ knowledge, this is the first study investigating whether crude oil exposure in a P*_*O*_* generation could influence offspring phenotype in birds.

### Direct Effects of Crude Oil Exposure in the P_0_ Parental Generation

Exposure to crude oil in avian species induces life-threatening effects. Dermal exposure to crude oil leads to impairment of thermoregulatory capacities, decrease in buoyancy and changes in navigational abilities in cormorants, gulls, and western sandpipers (Maggini et al., 2017a; Harr et al., 2017c). In addition, dietary exposure to crude oil induced loss of body mass, loss of appetite, loss of weight, and hepatic and renal diseases in sanderlings and semipalmated plovers, cormorants, and American kestrels (Pattee and Franson, 1982; Burger, 1997; Harr et al., 2017a, b). In contrast, in the present study neither of the dietary treatments induced mortality in the experimental groups. The actual concentration of oil load may have contributed to these differences. Noteworthy is that at the concentration levels of crude oil used in this study, which are lower than concentrations used in other studies (for a review see King et al., 2020), we did not observe changes in appetite or loss of body mass in the exposed groups. However, we did observe diarrheic feces in the High-oil-exposed group beginning on the third week of exposure, suggesting a potential disruption of the hepatic function and the digestive tract integrity (Leighton, 1986). Our observations align with previously reported results where watery diarrhea was observed in cormorants and gulls after dietary exposure to crude oil exposure (Leighton, 1986; Cunningham et al., 2017; Harr et al., 2017a).

Studies investigating the effects of crude oil exposure on the oxygen consumption and carbon dioxide production in adult birds are scarce and have been overshadowed by those focusing on the effects of thermoregulatory variables. In the present study $\dot{\text{V}}$O_2_ and $\dot{\text{V}}$CO_2_ were not affected by any of the dietary crude oil treatments in the adult populations. These results align with the results previously reported in nestling adult Storm-petrels (*Oceanodroma leucorhoa*; Butler et al., 1986). Respiratory distress due to crude oil exposure, exhibited as changes in ventilation rates, has been reported previously in birds (Mazet et al., 2002). In contrast, we found no differences induced by the dietary oil treatments in any measured ventilatory variables (Figure 2). This result suggests that the levels of dietary crude oil exposures used did not compromise resting metabolic rates at the organismal level. However, our measurements were performed in standard controlled conditions (i.e., room temperature, food, and water *ad libitum*) where the adults birds where not exposed to additional stressors beyond oil exposure. Consequently, possible alterations in metabolic rates induced by dietary oil exposure could occur when the animals face constantly changing environments such as natural daily changes of environmental temperature.

We additionally analyzed the effects of dietary crude oil exposure on blood parameters (Table 1). From the 9 measured blood chemistry and hematology variables, we found differences between experimental groups just in three of them: arterial PO_2_, PCO_2_, and SO_2_. Arterial PO_2_ and SO_2_ were higher in the Control group in comparison with the Low and High oil-exposed groups and PCO_2_ was higher in oil-exposed groups compared with the Control group. However, interpretation of these results is complicated by the fact that blood pH, HCO3-, and lactate did not change (Table 1). In addition, it is widely known that anesthesia can induce respiratory depression and bradycardia in birds, however, at least to the authors knowledge, virtually nothing is known about if crude oil exposure may increase susceptibility to anesthesia, and if those effects could have been reflected in the results. Furthermore, studies implementing surgical techniques are warranted to explore these possibilities.

Additionally, we found no differences in [Hb] and Hct values between experimental groups. These results contrast with oil-induced changes in blood parameters, accompanied with hemolytic anemia, caused by ROS oxidative damage and the subsequent lysis of RBC in chickens, American oystercatchers, black skimmers, brown pelicans, cormorants, and great egrets (Couillard and Leighton, 1991; Harr et al., 2017a; Fallon et al., 2018). Overall, these results suggest a possible impairment of blood oxygen transport by crude oil exposure. However, to test this hypothesis, future studies focused on determining the molecular implications of oil exposure in blood and hemoglobin, including specific mechanisms of O_2_-binding affinity and genetic control on these variables are warranted.

Heart mass of the exposed adults was smaller than in the Control group, while kidney mass was larger in the exposed groups (Table 2). The heart and the kidney were the only organs in which we observed differences between experimental groups. Crude oil exposure in laughing gulls (*Leucophaeus atricilla*) and in double crested cormorants (*Phalacrocorax auritus*) led to increased loss of structural integrity and cardiac damage (Horak et al., 2017; Harr et al., 2017b). Therefore, it is possible that the loss of heart mass observed in this study in both of the oil exposed groups could be the result of similar effects. Additionally to effects in heart mass, the decreased kidney mass in oil exposed quail observed in this study suggest that kidney tissue was damaged as reported in oil exposed Western (*Calidris mauri*), and double crested cormorants, which exhibited cell hypertrophy, including adrenal gland hypertrophy and inflammatory lesions in renal tubules/ductules (Bursian et al., 2017b; Harr et al., 2017b). Future histological studies are warranted to further explore these possibilities.

Surprisingly, in the present study we found no differences in liver mass between experimental groups. The liver is one of the most important organs for metabolic regulation and detoxification during toxicant exposure and increases in liver mass in response to crude oil ingestion have been reported previously (Dean et al., 2017; Horak et al., 2017; Harr et al., 2017b). Histopathological studies would reveal if dietary crude oil exposure at the levels used in this study induced effects at a deeper level (e.g., necrosis, neoplasia). Most of the organ masses, including that of the liver, differed between sex within the groups, a reflection of sexual dimorphism evident in body mass differences between sexes (Table 2). Overall, the results from the parental population suggest that 3 weeks of dietary exposure to specific crude oil doses is sufficient to generate significant differences in adults, helping to define further investigations. Additionally, we found inter-sex differences in the measured respirometry variables, highlighting the importance of considering sex as a factor when designing experimental protocols.

### Transgenerational Developmental Effects of Crude Oil in the F_1_ Generation

An organism’s developmental trajectory depends upon both its own genetic instructions as well as phenotypes deriving from non-genetic inheritance related to parental environmental experiences (Burggren and Dubansky, 2018). Crude oil exposure and its components has been shown to induce transgenerational effects in fish (Corrales et al., 2014; Bautista and Burggren, 2019). However, the study of transgenerational effects of crude oil exposure in avian species has received little attention (Paiva et al., 2018; King et al., 2020). To address this, we demonstrated some altered phenotypes related to thermoregulation, respiratory physiology, and gas exchange in non-exposed eggs and hatchlings quail obtained from the parental adult exposed groups.

Measurements of egg mass, length, and width revealed no differences between experimental groups induced by the parental dietary exposure (Figure 3). However, at the physiological level egg water loss was increased in eggs obtained from the Low and the High-level of oil-exposed parental groups when compared to controls (Figure 3D). Maintenance of humidity and temperature during egg incubation is of critical importance for eggshell quality and for the calcification process during the egg’s passage through the female bird’s oviduct, thus affecting survival during early development in the embryos (Yang et al., 2010; Branum et al., 2016). Our results showing modified water vapor flux across the eggshell in the High oil level exposed group suggests a functional disturbance of the female oviduct due to stress that potentially arose from dietary oil exposure. These data are aligned with reported reduction in egg production, yolk deposition, eggshell thinning, and fertilization reported after oil exposure in several bird species (Lewis and Malecki, 1984; Leighton, 1993; Stubblefield et al., 1995; Azkona et al., 2006; Alonso-Alvarez et al., 2007). The present study did not analyze changes in eggshell thickness, nor pore size in the eggshells. However, these factors are likely to be a cause of the increased Wl_*oss*_ in eggs from High-oil-exposed adults. Furthermore, embryonic survival could be compromised in the face of fluctuating environmental conditions that challenge the maintenance of adequate humidity and embryonic oxygen consumption in the wild (King et al., 2020). Therefore, further investigations to elucidate if dietary exposure to crude oil at the levels used in this study will induce similar effects in fecundity and eggshell characteristics are warrant.

The hatchlings of all experimental quail groups exhibiteda phenotype typical of precocial avian hatchlings characterized by insufficient thermal insulation to efficiently maintain metabolically generated heat, and that is reflected in the decrease in body temperature as ambient temperature decreased (Figure 5). However, the hatchings of the High oil-exposed parental population exhibited statistically lower body temperatures compared to the Control and the Low groups at almost all ambient temperatures. This result suggests that insulation and heat retention capacities in the F*_1_* offspring were potentially impaired as a result of parental oil exposure. Therefore, it is possible that natural populations in the wild may experience increased risk of mortality. This is a significant finding, because oil exposure through fouling can reduce the ability of adult birds to maintain body temperature. Future experiments will be necessary to determine if the impaired thermoregulatory abilities evident in hatchlings are retained into adulthood. Impaired thermoregulatory capacities may be result from the disruption of hormonal signaling (thyroid hormones) due to oil exposure. These effects have been reported previously in mammals (Fowles et al., 2016).

$\dot{\text{V}}$O2, $\dot{\text{V}}$CO2, and RER did not differ significantly at any ambient temperature or between any experimental group (Figure 7). These results suggest that the parental oil-exposure did not affect the offspring’s capacity to maintain whole organismal metabolic variables that reflect the integrated function of the respiratory and cardiovascular system. Therefore, we investigated whether these results were due to the adjustment of subordinate components.

At the most challenging temperatures of the cooling experimental protocol (19–22°C), *f* was unaffected, but V_*T*_ and thus $\dot{\text{V}}$_*E*_ were significantly elevated in all populations, with hatchlings from High oil-exposed parents showing the highest increase (Figure 7). Regarding the efficiency of gas exchange, air convection requirement increased at the two lower temperatures, but was unaffected by parental history. This effect could be the result of different scenarios, for example reduced ability of the lungs to acquire oxygen at the lowest ambient temperatures. However, this possibility is contradicted the decrease in body temperature would result in the left shift of the oxygen equilibrium curve, increasing hemoglobin-oxygen binding affinity and aiding pulmonary oxygen uptake. Clearly, further studies are required to determine the mechanism underlying the decrease in gas exchange effectiveness at the lower temperature, and in particular why this was greatest in hatchlings from oil-exposed parents.

To our knowledge, this is the first study investigating how dietary crude oil exposure in the P*_*O*_* influences offspring’s physiological and morphological phenotypes in developing birds that, themselves, had not been directly exposed to oil. Overall, our findings of relatively few physiological effects (except involving thermoregulation) are surprising, given that embryonic developmental stages represent periods of high sensitivity to effects elicited by the parental dietary oil exposure, a characteristic also seen in other organisms (Auge et al., 2017; Burggren and Dubansky, 2018; Bautista et al., 2020). Notably, we incubated the eggs in strictly controlled conditions, and so it is possible that some effects in early hatchling development were attenuated or unnoticed. Thus, future studies that consider “wild” incubation patterns are needed to explore and reveal the possible impairment of thermogenic capacities during early embryonic development.

Finally, it is widely accepted for birds that the development of the subsequent generation is affected by the yolk components deposited by the mother (Ho and Burggren, 2010; Guerrero-Bosagna et al., 2018; Jenni-Eiermann et al., 2020). That there were no PAHs detected in the offspring eggs from the experimental parental groups in the present study suggests that there were no direct effects of PAHs in the offspring. However, it is possible that components other than the analyzed PAHs may be inherited from the mother through the yolk (Hagmayer et al., 2018). Molecular studies focused on identifying the presence and the inheritance of epigenetic markers are needed to determine the mechanism underlying the inheritance of altered phenotypes in the F*_1_* generation.

## Conclusion and Future Directions

Despite the devastating effects of external exposure to petroleum in bird populations, only a few studies have attempted to understand the effects of different routes of exposure and their consequences. Although dietary exposure to crude oil may not lead to increased mortality in the exposed birds, it may affect their physiological functions, as well as the normal development of their offspring. The present study has demonstrated that, indeed, parental exposure to crude oil via diet in the king quail may impact embryonic development of their offspring, even when they themselves have not been directly exposed to oil. To better understand the transgenerational impacts of environmental stressors such as crude oil, future studies should focus on exposure effects of the offspring throughout their life span, and also track potential phenotypic effects across multiple generations. In addition, as responses at the whole organismal level may not be complementary with molecular responses, a multi-level approach aimed to decipher the molecular basis of these effects will provide more complete answers.

## Data Availability Statement

The datasets presented in this study can be found in online repositories. The names of the repository/repositories and accession number(s) can be found below: https://data.gulfresearchinitiative.org/data/R6.x804.000:0025.

## Ethics Statement

The animal study was reviewed and approved by Institutional Animal Care and Use Committee.

## Author Contributions

NB: conceptualization, data acquisition, processing, analysis, and interpretation, performance of the experiments, drafting of the manuscript, and editing. LA-S: conceptualization, data acquisition, performance of the experiments, data acquisition and interpretation, and manuscript editing. ED: equipment, data interpretation, and manuscript editing. WB: conceptualization, data interpretation, manuscript editing, and Funding. All authors contributed to the article and approved the submitted version.

## Conflict of Interest

The authors declare that the research was conducted in the absence of any commercial or financial relationships that could be construed as a potential conflict of interest.

## Abbreviations

dph: days post-hatching
*f*: breathing frequency
F_*E*_O_2_: excurrent O_2_ fraction of dry gas
F_*I*_O_2_: incurrent O_2_ fraction of dry gas
F_*E*_CO_2_: excurrent CO_2_ fraction of dry gas
F_*I*_CO_2_: incurrent CO_2_ fraction of dry gas
$\dot{\text{V}}$_*I*_: incurrent flow rate
T_*a*_: air temperature
T_*b*_: body temperature
$\dot{\text{V}}$O_2_: oxygen consumption
$\dot{\text{V}}$CO_2_: carbon dioxide production
$\dot{\text{V}}$_*E*_: minute ventilation
$\dot{\text{V}}$_*E*_/$\dot{\text{V}}$O_2_: air convection requirement
V_*T*_: tidal volume
RER: respiratory exchange ratio
W_*los*__*s*_: Water loss across the eggshell.
