# Supplementary material for: Dietary Exposure to Low Levels of Crude Oil Affects Physiological and Morphological Phenotype in Adults and Their Eggs and Hatchlings of the King Quail (Coturnix chinensis)
Source: Front Physiol. 2021 Apr 9;12:661943. doi: 10.3389/fphys.2021.661943 (PMC8063051; doi:10.3389/fphys.2021.661943)
Supplement: Supplementary file 1 [file Data_Sheet_1.PDF]

Supplementary information

Fig. S1.

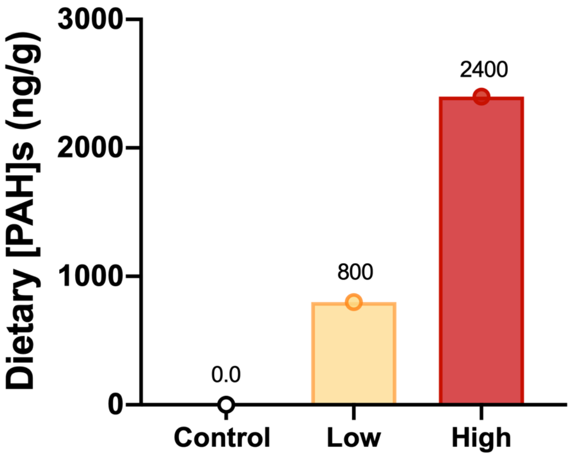

Fig. S1. Polycyclic aromatic hydrocarbon (PAH) concentration in dietary treatments for the control, low, and medium oil level exposed group. Representative samples of each treatment diet were analyzed by ALS Environmental (ALS Environmental, Kelso, WA, USA)

Table S1. List of components and nominal concentrations (ng/g and mg/kg) in eggs obtained from each parental group (control, Low, and High level exposed oil). ND= not detected.

**Component**	Control	Low (800 ng/g)	High (2400 ng/g)	Blank	**Units**
Naphthalene	0.86	ND	0.99	ND	ug/Kg
2-Methylnaphthalene	ND	ND	0.79	ND	ug/Kg
1-Methylnaphthalene	ND	ND	ND	ND	ug/Kg
C2-Naphthalenes	ND	ND	ND	ND	ug/Kg
C3-Naphthalenes	ND	ND	ND	ND	ug/Kg
C4-Naphthalenes	ND	ND	ND	ND	ug/Kg
Biphenyl	ND	ND	ND	ND	ug/Kg
Acenaphthylene	ND	ND	ND	ND	ug/Kg
Dibenzofuran	ND	ND	ND	ND	ug/Kg
Acenaphthene	ND	ND	ND	ND	ug/Kg
Fluorene	ND	ND	ND	ND	ug/Kg
C1-Fluorenes	ND	ND	ND	ND	ug/Kg
C2-Fluorenes	ND	ND	ND	ND	ug/Kg
C3-Fluorenes	ND	ND	ND	ND	ug/Kg
Dibenzothiophene	ND	ND	ND	ND	ug/Kg
C1-Dibenzothiophenes	ND	ND	ND	ND	ug/Kg
C2-Dibenzothiophenes	ND	ND	ND	ND	ug/Kg
C3-Dibenzothiophenes	ND	ND	ND	ND	ug/Kg
Phenanthrene	ND	ND	ND	ND	ug/Kg
Anthracene	ND	ND	ND	ND	ug/Kg
C1-Phenanthrenes/Anthracenes	ND	ND	ND	ND	ug/Kg
C2-Phenanthrenes/Anthracenes	ND	ND	ND	ND	ug/Kg
C3-Phenanthrenes/Anthracenes	ND	ND	ND	ND	ug/Kg
C4-Phenanthrenes/Anthracenes	ND	ND	ND	ND	ug/Kg
Fluoranthene	ND	ND	ND	ND	ug/Kg
Pyrene	ND	ND	ND	ND	ug/Kg
C1-Fluoranthenes/Pyrenes	ND	ND	ND	ND	ug/Kg
Benz(a)anthracene	ND	ND	0.67	ND	ug/Kg
Chrysene	ND	ND	ND	ND	ug/Kg
C1-Chrysenes	ND	ND	ND	ND	ug/Kg
C2-Chrysenes	ND	ND	ND	ND	ug/Kg
C3-Chrysenes	ND	ND	ND	ND	ug/Kg
C4-Chrysenes	ND	ND	ND	ND	ug/Kg
Benzo(b)fluoranthene	ND	ND	ND	ND	ug/Kg
Benzo(k)fluoranthene	ND	ND	ND	ND	ug/Kg
Benzo(e)pyrene	ND	ND	ND	ND	ug/Kg
Benzo(a)pyrene	ND	ND	ND	ND	ug/Kg
Perylene	ND	ND	ND	ND	ug/Kg
Indeno(1,2,3-cd)pyrene	ND	ND	ND	ND	ug/Kg
Dibenz(a,h)anthracene	ND	ND	ND	ND	ug/Kg
Benzo(g,h,i)perylene	ND	ND	ND	ND	ug/Kg
Fluorene-d10	59	56	60	68	PERCENT
Fluoranthene-d10	76	72	76	84	PERCENT
Terphenyl-d14	80	75	80	81	PERCENT

frontiers
